# Recent horizontal transfer, functional adaptation and dissemination of a bacterial group II intron

**DOI:** 10.1186/s12862-016-0789-7

**Published:** 2016-10-20

**Authors:** Félix LaRoche-Johnston, Caroline Monat, Benoit Cousineau

**Affiliations:** Department of Microbiology and Immunology, Microbiome and Disease Tolerance Centre (MDTC), McGill University, Montréal, Québec H3A 2B4 Canada

**Keywords:** *Enterococcus faecalis*, *Lactococcus lactis*, Ll.LtrB, Ef.PcfG, Lateral transfer, Intron evolution, Intron mobility, Intron dissemination, Conjugation

## Abstract

**Background:**

Group II introns are catalytically active RNA and mobile retroelements present in certain eukaryotic organelles, bacteria and archaea. These ribozymes self-splice from the pre-mRNA of interrupted genes and reinsert within target DNA sequences by retrohoming and retrotransposition. Evolutionary hypotheses place these retromobile elements at the origin of over half the human genome. Nevertheless, the evolution and dissemination of group II introns was found to be quite difficult to infer.

**Results:**

We characterized the functional and evolutionary relationship between the model group II intron from *Lactococcus lactis*, Ll.LtrB, and Ef.PcfG, a newly discovered intron from a clinical strain of *Enterococcus faecalis*. Ef.PcfG was found to be homologous to Ll.LtrB and to splice and mobilize in its native environment as well as in *L. lactis*. Interestingly, Ef.PcfG was shown to splice at the same level as Ll.LtrB but to be significantly less efficient to invade the Ll.LtrB recognition site. We also demonstrated that specific point mutations between the IEPs of both introns correspond to functional adaptations which developed in *L. lactis* as a response to selective pressure on mobility efficiency independently of splicing. The sequence of all the homologous full-length variants of Ll.LtrB were compared and shown to share a conserved pattern of mutation acquisition.

**Conclusions:**

This work shows that Ll.LtrB and Ef.PcfG are homologous and have a common origin resulting from a recent lateral transfer event followed by further adaptation to the new target site and/or host environment. We hypothesize that Ef.PcfG is the ancestor of Ll.LtrB and was initially acquired by *L. lactis*, most probably by conjugation, via a single event of horizontal transfer. Strong selective pressure on homing site invasion efficiency then led to the emergence of beneficial point mutations in the IEP, enabling the successful establishment and survival of the group II intron in its novel lactococcal environment. The current colonization state of Ll.LtrB in *L. lactis* was probably later achieved through recurring episodes of conjugation-based horizontal transfer as well as independent intron mobility events. Overall, our data provide the first evidence of functional adaptation of a group II intron upon invading a new host, offering strong experimental support to the theory that bacterial group II introns, in sharp contrast to their organellar counterparts, behave mostly as mobile elements.

**Electronic supplementary material:**

The online version of this article (doi:10.1186/s12862-016-0789-7) contains supplementary material, which is available to authorized users.

## Background

Group II introns are phylogenetically widespread mobile retroelements present in bacteria, archaea, plant chloroplasts, and mitochondria of fungi and plants [1]. However, they are absent, and most likely functionally excluded [2] from the nuclear genomes of eukaryotes that are instead loaded with evolutionarily related intervening sequences called spliceosomal or nuclear introns [3].

Active group II intron ribonucleoprotein particles (RNPs) are composed of a large and highly structured RNA core associated with two copies of a multifunctional intron-encoded protein (IEP). Following transcription of the intron-interrupted gene, the IEP specifically binds the intervening sequence within the precursor mRNA transcript, assisting the intron to fold into its catalytically active tridimensional conformation. Self-splicing of the intron concurrently leads to the ligation of its flanking exons and the release of active RNPs. Both components of the intron RNPs intimately cooperate in the recognition and invasion of identical or similar sequences using the retrohoming or retrotransposition pathway, respectively [1, 4–7].

The architecture and genomic localization of group II introns are quite different depending on whether the host is bacterial or organellar, suggesting that they do not behave the same way in these distinct cellular environments [8–11]. Despite having often lost their IEPs, organellar group II introns are mostly splicing competent and usually interrupt housekeeping genes. These introns are thus more genomically stable and must splice efficiently to ensure adequate expression of the genes they interrupt. In contrast, bacterial group II introns are primarily truncated, inactivated, associated with other mobile genetic elements and located outside housekeeping genes. Taken together, these features suggest that organellar group II introns act almost solely as splicing ribozymes whereas bacterial group II introns behave mostly as mobile genetic elements, cycling through high rates of gain and loss [12]. Over evolutionary timescales, bacterial group II introns are believed to be deleterious to their host cells and to survive the streamlining pressure of purifying selection applied on bacterial genomes through repeated instances of extinction and recolonization, previously characterized as the selection-driven extinction model [13].

On a broad evolutionary perspective, group II introns are thought to have substantially shaped the origin and evolution of contemporary eukaryotic genomes. They are considered as the progenitors of the telomerase enzyme, the very abundant non-LTR retroelements and spliceosomal introns, and the nuclear intron splicing machinery, the spliceosome. Altogether, these presumed group II introns derivatives correspond to more than half of the human genome [1–3, 14, 15].

Despite their interesting history, the evolution and dissemination of group II introns was found to be quite difficult to study for several reasons [10, 11, 16]. Indeed, even though the retroelement ancestor hypothesis proposes a general pattern of coevolution between the intron RNA secondary structures and their related IEPs, several caveats remain which hamper the establishment of conclusive group II intron phylogenies [8, 17, 18]. First, both the RNA and protein components have several variant forms with different potential evolutionary histories [11]. Second, the use of amino acid sequences from the IEPs as a phylogenetic marker is quite limited due to the high level of sequence saturation and the potential bias of the amino acid composition depending on the bacterial host, leading to small signal-to-noise ratios and generating uncertainties about the inner nodes of the phylogenetic trees [10, 19]. Third, even if the size of the RNA component is significant (0.7 kb), it is only conserved at the secondary structure and thus evolves rapidly, leaving very limited primary sequence information as potential phylogenetic signal [20]. Finally, as retromobile elements that move between genetic locations and that can also be transferred amongst cells within and across species, direct evolutionary links between group II introns as well as with both the genes they interrupt and their host organisms are difficult to infer [21]. Therefore, definitive conclusions about the evolution and dissemination of group II introns can only be drawn by studying homologous introns that diverged relatively recently [11].

The presence of multiple classes of introns in a number of given bacterial species suggests that group II intron horizontal transfer is quite common [22]. Accordingly, the majority of functional bacterial group II introns are found associated with other mobile genetic elements, such as conjugative plasmids, transposons, and IS elements [11, 21]. However, only a limited number of natural horizontal transfer events have been conclusively demonstrated, and in every case the precise origin of the transferred intron was impossible to infer [11, 16, 22–24]. Previous studies have shown that Ll.LtrB, the model group II intron from the gram-positive bacterium *Lactococcus lactis*, is able to invade conserved loci within orthologous genes in transconjugant bacterial strains by both retrohoming and retrotransposition, following the intra- (*L. lactis* to *L. lactis*) and inter-species (*L. lactis* to *Enterococcus faecalis*) transfer of its host conjugative elements [25–28].

Here we describe the functional and evolutionary relationship between Ef.PcfG, a newly discovered group II intron from a clinical strain of *E. faecalis* (SF24397), and the model group II intron from *Lactococcus lactis*, Ll.LtrB. Overall, our data support the hypothesis that Ef.PcfG is ancestral to Ll.LtrB and was acquired by *L. lactis*, most likely by conjugation, through a single horizontal transfer event. Repeated instances of conjugation-based horizontal transfer and independent intron mobility events led to the current colonization status of *L. lactis*. We also show for the first time the functional adaptation of a group II intron following its acquisition by horizontal transfer, providing strong experimental support to the theory that group II introns behave mostly as mobile elements in bacterial cells.

## Results

### A clinical isolate of *Enterococcus faecalis* contains a functional group II intron closely related to Ll.LtrB from *Lactococcus lactis*

We identified by sequence comparison a novel group II intron in the SF24397 strain of *E. faecalis* isolated from the urine sample of a patient in Michigan, USA [29]. The intron was found on a large contig within a region of high sequence similarity to the *E. faecalis* pTEF2 conjugative plasmid [30]. It interrupts a relaxase gene, *pcfG*, at the exact same conserved position Ll.LtrB interrupts the *ltrB* relaxase gene in *L. lactis* [28, 31]. Because of its origin (*E. faecalis*) and genetic location (*pcfG*) this novel group II intron and its intron-encoded protein were named Ef.PcfG and IepG respectively.

**Fig. 1:**
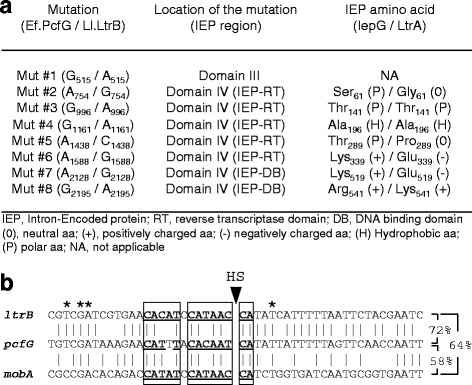
Comparison between the Ef.PcfG and Ll.LtrB group II introns and various relaxase genes from *L. lactis* and *E. faecalis*. **a** Description and location of the eight point mutations (Mut #1-Mut #8) that distinguish Ef.PcfG from Ll.LtrB within pRS01. All mutations except one (Mut #1, domain III) are located in domain IV within the intron-encoded protein (IEP) gene. Five mutations (Mut #2 to Mut #6) are located in the reverse transcriptase domain (IEP-RT) while two (Mut #7, Mut #8) are within the DNA binding domain (IEP-DB) of the IEP. Among the mutations located within the IEP, five are missense (Mut #2, Mut #5 to Mut #8) while the other two are silent (Mut #3, Mut #4). The nucleotide (nt) and amino acid (aa) numberings are in reference to the first nt of the intron (2492 nt) and the first aa of the IEP (599 aa) respectively. **b** Sequence alignement of the intron insertion sites in various relaxase genes from *L. lactis* (*ltrB*) and *E. faecalis* (*pcfG* and *mobA*). The intron insertion site or homing site (HS) (black arrowhead) and the percentage of homology between the three sequences are depicted. The IBS1, IBS2 and ∂’ sequences are boxed and the nts that are complementary to the EBS1, EBS2 and ∂ sequences are bolded and underlined. Nts of *ltrB*-HS that are known to interact with LtrA are denoted by an asterisk [50]

Ef.PcfG is almost identical to Ll.LtrB (99.7 %), the model group II intron from the *L. lactis* conjugative plasmid, pRS01 [32], exhibiting only eight point mutations out of a total of 2492 nts (Fig. 1). The majority of the mutations (7/8) are located in domain IV, within the IEP (Mut #2-Mut #8), while one mutation (Mut #1) is located within the ribozyme portion of the intron in a bulged region of domain III. Five of the mutations in domain IV are missense mutations leading to amino acid changes in either the reverse transcriptase (IEP-RT) or the DNA binding (IEP-DB) domain of the IEP (Fig. 1a).

Plasmid isolation from *E. faecalis* SF24397 revealed the presence of two resident plasmids: a large pTEF2-like plasmid harboring the interrupted *pcfG* relaxase gene and the pEF1071 conjugative plasmid (9328 bp), containing an uninterrupted relaxase gene called *mobA* [33]. To study Ef.PcfG in a more convenient genetic environment we generated the SF24397ΔpEF1071 strain by curing the pEF1071 plasmid with novobiocin [34].

**Fig. 2:**
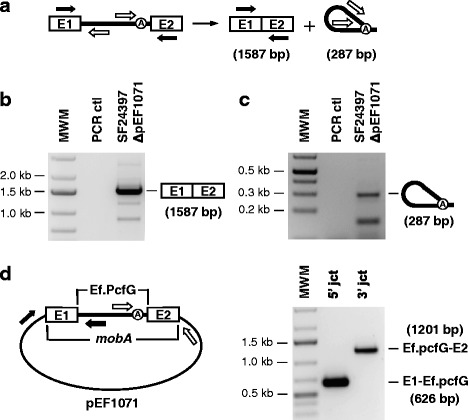
Splicing and mobility of the Ef.PcfG intron in its native environment. **a** Schematic of the group II intron splicing pathway. Position of the primers (Additional file 1: Table S2) used to amplify ligated exons (E1/E2) (black arrows, 1587 bp) and the intron splice junction (open arrows, 287 bp) by RT-PCR is depicted. RT-PCR amplifications of ligated exons **b** and of intron splice junctions **c** were performed on total RNA extracts from *E. faecalis* (SF24397ΔpEF1071). The RT-PCR amplicons corresponding to *pcfG* ligated exons (*b*, 1587 bp) and Ef.PcfG spliced junction (*c*, 287 bp) were excised and directly sequenced. **d** Mobility efficiency of Ef.PcfG to the relaxase *mobA* gene (E1/E2) on pEF1071. Position of the primers (Additional file 1: Table S2) used to amplify the 5’ (black arrows, 626 bp, E1-Ef.PcfG) and 3’ (open arrows, 1201 bp, Ef.PcfG-E2) junctions of Ef.PcfG mobility products in *mobA* by PCR is depicted

To determine whether Ef.PcfG is functional in its native environment, we first assessed its ability to splice in vivo by looking for both released intron and ligated exons by RT-PCR on *E. faecalis* (SF24397ΔpEF1071) total RNA extracts (Fig. 2a) [35, 36]. Sequencing of the major amplicons for both ligated exons and released intron showed that they correspond respectively to accurately joined *pcfG* exons (1587 bp) (Fig. 2b) and released intron lariats (287 bp) (Fig. 2c). Next, we wanted to examine if Ef.PcfG is mobile in *E. faecalis* (SF24397). We took advantage of the endogenous pEF1071 plasmid, which harbors an uninterrupted *mobA* relaxase gene of the same family as *ltrB* and *pcfG* [33, 37]. Even though the potential Ef.PcfG recognition site within *mobA* is not identical to its original recognition site in *pcfG* (Fig. 1b), we were able to dectect Ef.PcfG mobility products within *mobA* by PCR (Fig. 2d). Using two primer pairs, where one primer is specific for Ef.PcfG and the other specific for a plasmid sequence outside *mobA*, we amplified both the 5’ (E1-Ef.PcfG) (626 bp) and 3’ (Ef.PcfG-E2) (1021 bp) junctions of the intron mobility products (Fig. 2d). The sequence of both amplicons confirmed the precise insertion of Ef.PcfG within *mobA* at the expected position (Fig. 1b, arrowhead) [28].

These results reveal the presence of a novel group II intron, homologous to Ll.LtrB, in a clinical isolate of *E. faecalis* (SF24397). They also demonstrate that the interrupted *pcfG* relaxase gene is expressed in *E. faecalis* and that following accurate splicing from its relaxase transcript, EF.PcfG can invade, at the exact same conserved position, the *mobA* relaxase gene present within the resident conjugative plasmid pEF1071.

### Ll.LtrB and Ef.PcfG can recognize and invade each other’s homing sites

Several facts support the hypothesis of a recent event of group II intron horizontal transfer between *E. faecalis* and *L. lactis*: i) the high degree of identity between Ll.LtrB and Ef.PcfG (99.7 %) (Fig. 1a) compared to their full-length (61 %) or proximal flanking exons (72 %) (Fig. 1b); ii) both introns interrupt related relaxase genes at the exact same conserved position; iii) Ef.PcfG and Ll.LtrB are both splicing and mobile in their native environments; iv) Ll.LtrB is contained within different *L. lactis* conjugative elements previously shown to transfer to *E. faecalis* [25] while the *E. faecalis* pTEF2 plasmid, harboring Ef.PcfG, is closely related to the pheromone-sensitive pCF10 plasmid, that was shown to laterally transfer to *L. lactis* at very high efficiencies [38]; v) the *pcfG* gene of pCF10 was previously shown to be a functional target for Ll.LtrB in *E. faecalis* following inter-species conjugation experiments [28]. It was thus of interest to compare the functional characteristics of Ef.PcfG and Ll.LtrB to elucidate the evolutionary relationship between these two homologous bacterial group II introns.

**Fig. 3:**
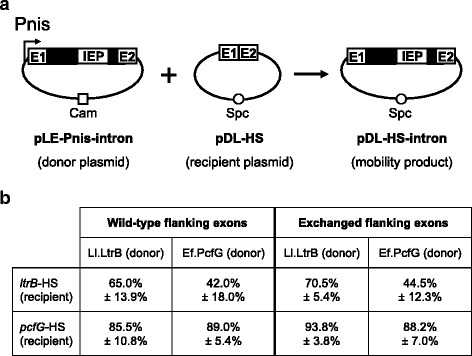
Mobility efficiency of Ef.PcfG and Ll.LtrB in *L. lactis*. **a** Schematic of the two-plasmid intron mobility assay. The assay consists of co-transforming both an intron donor and an intron recipient plasmid in *L. lactis* cells (NZ9800Δ*ltrB*) and monitoring for the appearance of intron mobility products. The intron donor plasmid harbors the *pcfG* or *ltrB* genes interrupted by their cognate or exchanged introns under the control of the nisin-inducible promoter (Pnis). The recipient plasmid contains either the *ltrB*-HS or the *pcfG*-HS (E1/E2). Upon nisin induction the intron can move from the donor to the recipient plasmid generating mobility products. Plasmid mixes (donor, recipient, mobility product) from independent mobility assays are recovered and the intron mobility efficiency is calculated as the percentage of recipient plasmids invaded by the intron (mobility product / (recipient + mobility product)). **b** Mobility efficiency of Ef.PcfG and Ll.LtrB at their own and each other’s homing sites. Two independent series of mobility assays were performed by expressing both introns flanked by either their wild-type (Wild-type flanking exons) or swapped exons (Exchanged flanking exons). Regardless of their flanking exons (Wild-type or exchanged), the mobility efficiency of both introns to the *pcfG*-HS is significantly higher (*p* < 0.05) than their efficiency towards the *ltrB*-HS while the mobility efficiency of Ll.LtrB to the *ltrB*-HS is significantly higher (*p* < 0.05) than Ef.PcfG. Each mobility efficiency value corresponds to the average of six independent mobility assays

Using a two-plasmid intron mobility assay (Fig. 3a) [39], we first measured and contrasted the efficiency of each intron to recognize and invade the recognition or homing site (HS) of both the *ltrB* and *pcfG* genes. The mobility assay consisted of co-transforming *L. lactis* with an intron donor plasmid, containing either the *ltrB*- (pLE-Pnis-*ltrB*E1-Ll.LtrB-*ltrB*E2) or *pcfG*- (pLE-Pnis-*pcfG*E1-Ef.PcfG-*pcfG*E2) interrupted gene downstream of the nisin-inducible promoter (Pnis), and an intron recipient plasmid harboring the HS of either the *ltrB* (pDL-*ltrB*-HS) or *pcfG* (pDL-*pcfG*-HS) gene (Fig. 3a) [39]. Intron mobility efficiency was subsequently calculated by patch hybridization as the proportion of intron recipient plasmids invaded by the intron [26].

The mobility efficiency of Ef.PcfG was found to be significantly higher at its own HS, *pcfG*-HS (89.0 %) than at the *ltrB*-HS (42.0 %) (Fig. 3b, Wild-type flanking exons). On the other hand, Ll.LtrB invaded the *pcfG*-HS (85.5 %) significantly more efficiently than its own recognition site, the *ltrB*-HS (65.0 %). Our data thus show that while the *ltrB*-HS is invaded significantly more efficiently by Ll.LtrB (65.0 % *vs* 42.0 %), both introns are capable to invade significantly more proficiently, and at similar levels, the *E. faecalis pcfG*-HS (65.0 % *vs* 85.5 % and 42.0 % *vs* 89.0 %).

Taken together, these results suggest that Ef.PcfG is ancestral to Ll.LtrB, and that following the invasion of the *ltrB* gene, Ef.PcfG adapted and evolved to mobilize significantly more efficiently to its new sequence environment, the *ltrB*-HS, without affecting its proficiency to invade its original recognition site, the *pcfG*-HS.

### The significant difference in mobility efficiency at the *ltrB*-HS between Ll.LtrB and Ef.PcfG is due to sequence variations within the introns

Having uncovered a significant difference in the capacity of both introns to invade the *ltrB*-HS, we sought to examine the cause of this difference. Sequence variations exist between Ef.PcfG and Ll.LtrB (Fig. 1a) and also among their flanking exons (Fig. 1b), both of which may potentially affect intron mobility efficiency [20]. We thus exchanged the introns between the interrupted *ltrB* and *pcfG* genes creating two new intron donor plasmids harboring Ll.LtrB flanked by the *pcfG* exons (pLE-Pnis-*pcfG*E1-Ll.LtrB-*pcfG*E2) and Ef.PcfG flanked by the *ltrB* exons (pLE-Pnis-*ltrB*E1-Ef.PcfG-*ltrB*E2) (Fig. 3b, Exchanged flanking exons).

Using our two-plasmid intron mobility assay (Fig. 3a) we found that the Ll.LtrB intron, despite being flanked by the *pcfG* exons, mobilized to its own HS with similar efficiency (70.5 % *vs* 65.0 %) (Fig. 3b). Likewise, the Ef.PcfG intron, interrupting the *ltrB* gene, mobilized to the *ltrB*-HS with comparable efficiency as wild-type Ef.PcfG (44.5 % *vs* 42.0 %). The same trend was observed for the mobility efficiency of both introns to the *pcfG*-HS.

Overall, mobility efficiencies of both introns are not significantly altered regardless of the nature of their flanking exons. Our data thus demonstrate that the difference in mobility efficiency to the *ltrB*-HS between Ll.LtrB and Ef.PcfG is not due to sequence variations amongst the flanking exons, but most likely due to the eight point mutations between the introns.

### The variation in mobility efficiency between Ef.PcfG and Ll.LtrB at the *ltrB*-HS is not due to changes in splicing efficiency

Two main factors can influence group II intron mobility efficiency: splicing or RNP release and homing site invasion. Following the observation that the difference in mobility efficiency between Ll.LtrB and Ef.PcfG to the *ltrB*-HS is most likely due to sequence variations within the introns, we wanted to first study if these point mutations affect splicing efficiency.

**Fig. 4:**
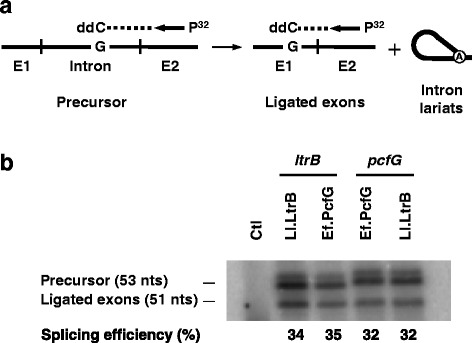
Splicing efficiency of Ef.PcfG and Ll.LtrB in *L. lactis*. **a** Group II intron splicing efficiency assessed by poisoned primer extension assay. This assay monitors splicing efficiency by comparing the relative abundance of precursor and ligated exons from total RNA extracts. A P^32^-labeled primer (Additional file 1: Table S2) complementary to exon 2 was extended from both the precusor and the ligated exons in the presence of a high concentration of ddCTP. Since the sequence of the two RNAs are different after the exon 2 junction the first G residue encountered is at a different distance from the primer generating differently sized bands for the precursor (53 nts) and the ligated exons (51 nts). **b** The splicing efficiency (%) of Ef.PcfG and Ll.LtrB was assessed from their wild-type and exchanged flanking exons and calculated as the relative intensity of the ligated exons and precursor bands (ligated exons/precursor + ligated exons)

To evaluate the splicing efficiency of our various intron constructs, we performed poisoned primer extension assays (Fig. 4a) [35, 39]. This assay compares the ratio of ligated exons to precursor mRNA from total RNA extracts. Since the sequence of the two RNAs are different after the exon 2 junction, the first G residue encountered is at a different distance from the primer, generating differently sized bands for the precursor and the ligated exons (Fig. 4a). Our data show that the splicing efficiency of Ll.LtrB and Ef.PcfG are almost identical, varying from 32 to 35 %, regardless of whether they are flanked by their cognate exons or not (Fig. 4b).

These results demonstrate that variations in splicing efficiency between Ll.LtrB and Ef.PcfG cannot account for the significant increase in mobility efficiency observed for Ll.LtrB at the *ltrB*-HS and rather suggest that Ll.LtrB is more proficient than Ef.PcfG during the invasion of the *ltrB*-HS.

### Some of the point mutations within Ef.PcfG increase its mobility efficiency to the *ltrB*-HS

Having demonstrated that the significant difference in mobility efficiency at the *ltrB*-HS between Ef.PcfG and Ll.LtrB arises from sequence variations within the introns and that these eight mutations (Fig. 1a, Mut #1 to Mut #8) do not affect splicing efficiency, we studied the individual effect of these mutations on the mobility efficiency of Ef.PcfG. Eight intron donor plasmids were engineered by site directed mutagenesis (pLE-Pnis-*pcfG*E1-Ef.PcfG-Mut #1-*pcfG*E2 to pLE-Pnis-*pcfG*E1-Ef.PcfG-Mut #8-*pcfG*E2) and co-transformed independently with pDL-*ltrB*-HS or pDL-*pcfG*-HS in *L. lactis*.

**Fig. 5:**
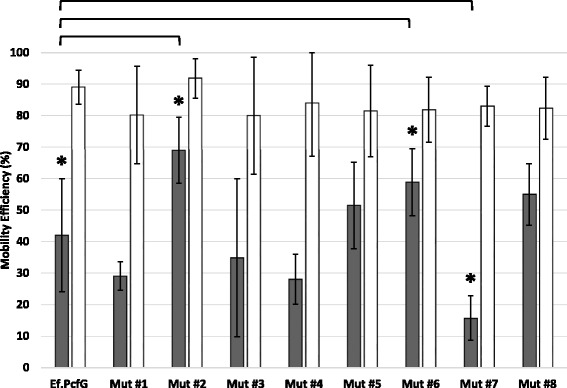
Graphical representation of the mobility efficiency of Ef.PcfG, flanked by its wild-type exons, to both the *ltrB*-HS (black bars) and the *pcfG*-HS (open bars). The eight point mutations between Ef.PcfG and Ll.LtrB were independently engineered within Ef.PcfG (Mut #1 to Mut #8) and their mobility efficiencies are compared to wild-type Ef.PcfG (*, *p* < 0.05). All mutants are significantly more efficient at homing to the *pcfG*-HS than the *ltrB*-HS (*p* < 0.05). Each mobility efficiency value corresponds to the average of six independent mobility assays

The mobility efficiency of these eight Ef.PcfG mutants was assessed using our two-plasmid mobility assay (Fig. 3a). Four mutations in domain IV (Mut #2, #5, #6, #8), within the IepG coding region, increased the mobility efficiency of Ef.PcfG to the *ltrB*-HS (Fig. 5, black bars), two of them significantly (Mut #2, #6). In contrast, three mutations did not significantly affect the mobility efficiency of Ef.PcfG to *ltrB*-HS (Mut #1, #3, #4). Mutation #1 is located in a bulged region of domain III not disrupting the predicted secondary structure or any of the previously identified long-range tertiary interactions [40]. Mutations #3 and #4, in domain IV, are silent and thus do not change the amino acid sequence of the IepG protein. Finally, mutation #7 lead to a significant decrease of the Ef.PcfG mobility efficiency to *ltrB*-HS. As expected, none of the eight point mutations significantly affected the mobility efficiency of Ef.PcfG to its own recognition site (pDL-*pcfG*-HS) (Fig. 5, open bars).

Taken together, these results demonstrate that half of the point mutations between Ef.PcfG and Ll.LtrB improved the mobility efficiency of Ef.PcfG to the *ltrB*-HS, two of them significantly. This supports the hypothesis that Ef.PcfG invaded the *ltrB*-HS and then functionally adapted following evolutionary pressure on the invasion efficiency of its new flanking exons.

### Full-length variants of Ll.LtrB in *L. lactis* share a conserved pattern of mutation acquisition

**Fig. 6:**
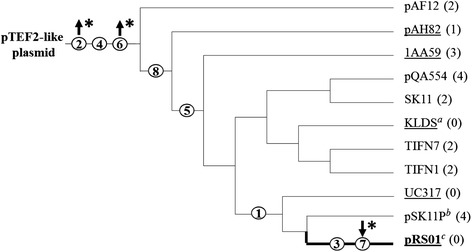
Dendrogram of mutation accumulation between Ef.PcfG from *E. faecalis* and the various Ll.LtrB introns identified from *L. lactis*. The root of the tree is depicted as the Ef.PcfG intron (pTEF-2-like plasmid), which likely disseminated throughout *L. lactis* following a single horizontal transfer event. The plasmids harboring the Ef.PcfG (pTEF-2-like plasmid) and Ll.LtrB (pRS01) introns discussed in this study are bolded. The eight point mutations (Mut #1 to Mut #8) that distinguish these two introns are shown in circles, with an asterisk adjacent to mutations conferring significant increases (*upwards arrow*) and decreases (*downwards arrow*) in mobility efficiency to the *ltrB*-HS (*p* < 0.05). Introns found in the chromosome are designated by the strain’s name, whereas introns found within plasmids are represented by the plasmid’s name. Strains or plasmids that are underlined belong to *L. lactis* subsp. *lactis*, whereas strains or plasmids that are not underlined belong to *L. lactis* subsp. *cremoris*. Thick branches of the dendrogram represent an insertion event into an HIHN-H relaxase motif, whereas thin branches represent an insertion event into an HLHN-H relaxase motif; the only exception being the intron present in the chromosome of SK11, which is likely a retrotransposition event into an ectopic site (cell surface protein). Numbers between parentheses denote the amount of additional mutations that distinguish a particular intron from Ef.PcfG in relationship to its position in the dendrogram. Three groups are present in the dendrogram which encompass a number of additional identical introns: Group (*a*) contains 6 additional introns (HP, TIFN5, TIFN6, FG2, B40, LMG6897), group (*b*) contains 3 additional introns (p3, SK110, AM2), and group (*c*) contains 4 additional introns (MG1363, pFI430, NZ9000, NCDO763) (Additional file 1: Table S3)

Having demonstrated that some point mutations within Ef.PcfG significantly improve its ability to invade the *ltrB*-HS, we analysed the distribution of the eight point mutations throughout all the homologous full-length group II introns present in *L. lactis* (Fig. 6). A total of 24 homologous full-length introns were thus identified, aligned and grouped together in clades based on nucleotide divergence from Ef.PcfG (Fig. 6). An additional group II intron, almost identical to Ef.PcfG (1 point mutation) was also identified in *E. faecalis* (533_EFLS). The presence of an additional nucleotide difference between this Ef.PcfG variant and Ll.LtrB (9 point mutations) suggests that the Ef.PcfG variant from SF24397 better represents the ancestral state of the intron that potentially colonized *L. lactis*.

All of the Ll.LtrB variants contain between 5 and 10 point mutations when compared to Ef.PcfG. The eight point mutations between Ef.PcfG and the Ll.LtrB model intron associated with the *L. lactis* pRS01 conjugative plasmid are found to progress throughout the dendrogram with all *L. lactis* introns containing mutations #2, #4, and #6 (Fig. 6). Although the distribution of the eight point mutations forms clear clades, introns throughout the dendrogram were found both in *L. lactis* subsp. *lactis* (Fig. 6, underlined) and *L. lactis* subsp. *cremoris* (Fig. 6, not underlined). These introns were also found interrupting different conserved functional motifs within relaxase genes, notably HLHN-H (Fig. 6, thin branches) and HIHN-H (Fig. 6, thick branches), with one instance of retrotransposition into an ectopic chromosomal site (SK11).

Overall, these results support the hypothesis that a single horizontal transfer event led to the introduction of an ancestral Ef.PcfG into *L. lactis*, whereby selective pressure caused the intron to adapt and evolve to its new host and/or sequence environment. This newly acquired intron then subsequently disseminated to other strains of *L. lactis*, most likely by conjugation, leading to the intra-species dispersal of group II introns in a new bacterial host.

## Discussion

In this study, we initially identified a functional group II intron, interrupting the *pcfG* relaxase gene of a clinical isolate of *E. faecalis*, which we named Ef.PcfG. This intron is homologous and almost identical to Ll.LtrB, the model group II intron from *L. lactis*, and was shown to splice and mobilize in its native host environment as well as in *L. lactis*. Interestingly, Ef.PcfG was found to splice at the same level as Ll.LtrB in *L. lactis* but to be significantly less efficient to invade the *ltrB*-HS. In contrast, both introns recognize the *pcfG*-HS significantly more proficiently, and at the same level. Finally, we identified, compiled and analyzed the homologous full-length variants of Ll.LtrB present in *L. lactis* and characterized the functional and evolutionary relationship between Ef.PcfG and Ll.LtrB from pRS01.

Overall, our data can be interpreted in different ways regarding both the direction and the order of the lateral transfer of the intron. The intron could have been transferred from *L. lactis* to *E. faecalis*, from *E. faecalis* to *L. lactis* or simply originated from a third partner. The Ll.LtrB variants present in *L. lactis* could also be the result of independent and recent horizontal transfer events rather than a single episode of horizontal transfer. Nevertheless, our results show that Ll.LtrB and Ef.PcfG are homologous and have a common origin resulting from a recent lateral transfer event followed by further adaptation to the new target site and/or host environment.

Even though we cannot rule out the alternative scenarios described above, our favored hypothesis supported by our data is that an ancestral Ef.PcfG colonized *L. lactis* from *E. faecalis*. Ef.PcfG would have most probably been transferred by conjugation relatively recently and initially invaded an orthologous relaxase gene of an *L. lactis* conjugative element. Following this single instance of horizontal transfer, the newly acquired intron would have adapted to its novel host and/or sequence environment in response to selective pressure specifically on target site invasion independently of splicing. Being associated with a conjugative element, the intron was further disseminated by conjugation between *L. lactis* strains and subspecies, invading the same highly conserved sequence motif present in two different catalytic histidine motifs of relaxase genes associated with various *L. lactis* conjugative elements.

Previous studies on the distribution of group II introns within bacterial populations revealed the presence of homologous group II introns in different bacterial strains and species [16, 22–24]. In these studies, however, the direction of the horizontal transfer, believed to be responsible for the dispersal of these introns, was impossible to infer. The observed genetic differences between these homologous retroelements thus provided no insights either into the nature of potential selective pressures affecting group II introns upon their colonization of a new sequence and/or host environment, or into how they would adapt to these hypothetical selective pressures. In contrast, our experimental data show that Ll.LtrB is significantly more efficient than Ef.PcfG to invade the *ltrB*-HS. This finding supports our hypothesis that the intron was horizontally transferred from *E. faecalis* to *L. lactis* and, combined with the fact that this difference in mobility efficiency is splicing-independent, indicates that the newly acquired intron adaptated to its new host and/or sequence environment following selective pressure specifically applied on target site invasion. The adaptation responsible for the increased efficiency of Ll.LtrB to invade the *ltrB*-HS was most likely recent enough that it did not affect its vestigial capacity to invade its ancestral recognition site, the *pcfG*-HS, at a higher efficiency. The analysis of the eight point mutations between Ef.PcfG and Ll.LtrB from pRS01 further supports our hypothesis. Mutations #2, #5, #6, and #8 all increase the Ef.PcfG mobility efficiency towards the *ltrB*-HS, mutations #2 and #6 significantly, while none of these individual mutations affect the mobility efficiency towards the *pcfG*-HS. The location of these amino acid substitutions in the RT (Mut #2, #5, #6) and DNA binding (Mut# 8) domains of IepG correlate with a splicing-independent increase in intron mobility.

Analysis of the homologous full-length Ll.LtrB variants in *L. lactis* revealed a pattern of mutation acquisition which, although not extensive enough to construct a conclusive phylogeny, enabled their grouping into clades based on the distribution of the eight point mutations between Ef.PcfG and Ll.LtrB from pRS01. Three mutations were found to be common to all of the analysed lactococcal introns (Mut #2, #4 and #6). Although it remains possible that these ubiquitous mutations only arose in the *E. faecalis* copy of Ef.PcfG after the horizontal transfer event occurred, two of them (Mut #2 and #6) lead to significant increases in mobility efficiency of Ef.PcfG towards the *ltrB*-HS. The appearance of these mutations could thus represent an adaptive response to a selective pressure on the mobility efficiency of the intron upon entering a new host and/or sequence environment. The third mutation common to all introns in *L. lactis* is a silent mutation (Mut #4), which accordingly showed no significant difference in the mobility efficiency of Ef.PcfG to either the *ltrB*-HS or *pcfG*-HS. It is thus impossible to determine whether this mutation was acquired independently in the *E. faecalis* copy of Ef.PcfG after the horizontal transfer event occurred, or whether it arose in the ancestor of all the Ll.LtrB variants. In either case, the ubiquitous presence of both beneficial mutations within all *L. lactis* introns supports the scenario of a single horizontal transfer event from *E. faecalis* to *L. lactis*, followed by subsequent dissemination and accumulation of additionnal independent mutations. This explanation offers a more parsimonious sequence of events than the alternative view involving multiple independent lateral transfer events and the independent acquisition of identical beneficial mutations by numerous introns.

Group II introns can theoretically be horizontally transferred by either invading a new site in a novel host or by the transfer of a previously interrupted gene. Nevertheless, we have found extensive evidence for independent intron mobility within *L. lactis* rather supporting the initial introduction of Ef.PcfG into the *ltrB*-HS by invasion of that new site rather than by acquisition of the interrupted gene. First, the stark contrast between the homology of Ef.PcfG and Ll.LtrB (99.7 %) compared with *pcfG* and *ltrB* (61 %) is a good indication that the initial introduction of Ef.PcfG into the *ltrB*-HS was by site-specific invasion. Second, aside the single exception of a retrotransposition event into a chromosomal gene coding for a cell surface protein (strain SK11), the homologous full-length introns were found to interrupt two different catalytic histidine motifs of relaxase genes: HLHN-H and HIHN-H [41]. Overall these data suggest that Ll.LtrB was acquired and disseminated through a series of horizontal transfer and independent mobility events.

The pTEF2-like element which harbors Ef.PcfG in SF24397 greatly resembles the pCF10 pheromone-sensitive plasmid, which has been shown to conjugate efficiently to *L. lactis* [38, 42]. This suggest that the initial introduction of Ef.PcfG into *L. lactis* was the result of a conjugative transfer. This is consistent with previous studies proposing that conjugation may play an important role for the spread of bacterial group II introns [9, 25–27]. Although pCF10 is not stably maintained in *L. lactis*, the transient introduction of a conjugative plasmid was shown to be sufficient for group II intron expression and the invasion of new loci in recipient cells [26]. On the other hand, lactococcal conjugative plasmids can also transfer to *E. faecalis*, allowing for a potential alternative scenario where an *L. lactis* conjugative plasmid could have been initially introduced into *E. faecalis*, invaded by Ef.PcfG, and then transferred back into *L. lactis* [43]. Our findings that various intermediates in the acquisition of the eight point mutations are found within various conjugative elements in either *L. lactis* subsp. *lactis* or *L. lactis* subsp. *cremoris*, which have no tendency to cluster in the dendrogram, suggests that upon arrival in *L. lactis*, additional conjugation-based horizontal transfer events lead to further dissemination of the intron.

Bacterial group II introns have been previously characterized as behaving more like retroelements than splicing-only introns [8]. The distribution of group II introns has largely supported this theory by finding markers underlining their behavior as transposable elements in constant movement, such as identical group II introns in different bacterial strains and species, unoccupied HSs in intron-containing bacteria, and numerous intron fragments alongside full-length copies [16, 22–24]. Group II introns have previously been shown to recognize new HSs more efficiently by modifying the exon binding sites 1 and 2 regions of their RNA component [44]. However, the mutations increasing the mobility efficiency of Ef.PcfG towards the *ltrB*-HS are located in IepG showing that group II introns are also able to adapt to new sequence and/or host environments by mutating their IEP. Our data thus demonstrate for the first time the functional adaptation of a group II intron following its acquisition by horizontal transfer, providing strong experimental support to the theory that group II introns behave mostly as mobile elements in bacteria.

## Conclusions

This work shows that Ll.LtrB and Ef.PcfG are homologous and have a common origin resulting from a recent lateral transfer event followed by further adaptation to the new target site and/or host environment. We hypothesize that Ef.PcfG is the ancestor of Ll.LtrB and was initially acquired by *L. lactis*, most probably by conjugation, via a unique event of horizontal transfer. Strong selective pressure on homing site invasion efficiency then led to the emergence of beneficial point mutations in the IEP, enabling the successful establishment and survival of the group II intron in its novel lactococcal environment. The current colonization state of Ll.LtrB in *L. lactis* was probably later achieved through recurring episodes of conjugation-based horizontal transfer as well as independent intron mobility events. Overall, our data provide the first evidence of functional adaptation of a group II intron upon invading a new host, offering strong experimental support to the theory that bacterial group II introns, in sharp contrast to their organellar counterparts, behave mostly as mobile elements.

## Methods

### Bacterial strains and plasmids

*Lactococcus lactis* strain NZ9800Δ*ltrB* (Tet^R^) [7] was grown in M17 media supplemented with 0.5 % glucose (GM17) at 30 °C without shaking. The *Escherichia coli* strain DH10β was grown in LB at 37 °C with shaking. The two strains of *Enterococcus faecalis*, SF24397 (Erm^R^/Gen^R^) [29] and SF24397ΔpEF1071 (Erm^R^) (this study), were grown in BHI at 37 °C without shaking. To generate the SF24397ΔpEF1071 strain, the resident plasmid pEF1071 was cured from SF24397 by treatment with Novobiocin (15 μg/μl) [34]. Antibiotics were used at the following concentrations: chloramphenicol (Cam^R^), 10 μg/ml; spectinomycin (Spc^R^), 300 μg/ml; erythromycin (Erm^R^), 300 μg/ml.

Plasmids and primers used in this study are listed in Additional file 1: Table S1 and S2 respectively. pLE-Pnis-*ltrB*E1-Ll.LtrB-*ltrB*E2 and pLE-Pnis-*pcfG*E1-Ef.PcfG-*pcfG*E2 were constructed by first cloning (BamHI) the nisin-inducible promoter (Pnis) within the shuttle plasmid pLE1 (Cam^R^) [45, 46]. The *ltrB* and *pcfG* genes interrupted by their respective introns, Ll.LtrB and Ef.PcfG, were then cloned (NotI) downstream of Pnis. The pLE-Pnis-*pcfG*E1-Ll.LtrB-*pcfG*E2 and pLE-Pnis-*ltrB*E1-Ef.PcfG-*ltrB*E2 were generated by swapping a restriction fragment (BsrGI/BsiWI) that contains the eight point mutations between both plasmids. The pLE-Pnis-*pcfG*E1-Ef.PcfG-*pcfG*E2-Mut #1-Mut #8 plasmids were generated independently by site-directed mutagenesis (New England Biolabs® Q5® Site-Directed-Mutagenesis Kit) (primers in Additional file 1: Table S2). The intron recipient plasmid pDL-*ltrB*-HS contains a 271 bp fragment (HindIII) of the *ltrB* relaxase gene, encompassing the native Ll.LtrB homing site, inserted within the pDL278 plasmid (SmaI) [46]. Similarly, the pDL-*pcfG*-HS plasmid harbors a 602 bp PCR amplicon (AclI) of the *pcfG* relaxase gene (primers in Additional file 1: Table S2), cloned into pDL278 (SmaI).

### Two-plasmid intron mobility and patch hybridization assays

To assess Ef.PcfG and Ll.LtrB mobility efficiency to both the *ltrB*-HS and *pcfG*-HS, NZ9800Δ*ltrB* cells containing an intron donor and an intron recipient plasmid were induced for intron expression with nisin as previously described [39]. Plasmid mixes (donor, recipient, mobility product) were extracted and electroporated into *E. coli* strain DH10β, which were then plated on LB/Spc plates to select for pDL-based plasmids (recipient plasmids and mobility products). The percentage of mobility efficiency was then obtained by patching 100 isolated colonies onto a new LB/Spc plate. Patches were lifted on a Hybond-N nylon membrane (Amersham™) and screened with a P^32^-labelled intron specific probe (Additional file 1: Table S2) to reveal intron mobility events. Mobility efficiency was then calculated as a percentage of positive hybridization events out of 100 colonies [39]. Statistical significance was calculated using an unpaired Student’s *T*-test, with *p* < 0.05.

### RNA extraction, RT-PCR, PCR and poisoned primer extension

Total RNA was isolated from SF24397ΔpEF1071 and NZ9800Δ*ltrB* harboring various plasmid constructs as previously described [47]. RT-PCR reactions [47] and poisoned primer extensions [39] were performed on total RNA preparations of SF24397ΔpEF1071 and NZ9800Δ*ltrB* harboring various intron constructs, respectively (primers in Additional file 1: Table S2). PCR amplifications of the 5’ and 3’ mobility junctions of Ef.PcfG within *mobA* of pEF1071 (primers in Additional file 1: Table S2) were performed on a plasmid preparation from *E. faecalis* (SF24397).

### Dendrogram of group introns present in *L. lactis*

A BLASTN search was performed using the Ef.PcfG intron as the query throughout all *L. lactis* genome and plasmid sequences available in the NCBI database. The homologous full-length introns (2492 nt) were compiled and aligned using the Clustal Omega alignment software [48]. The introns were organized into a Neighbour-joining tree without distance corrections, which was then exported to the interactive tree of life software (iTOL) for visualization [49].

## Additional file

Additional file 1: Table S1. Plasmids used in this Study. **Table S2.** Primers used in this Study. **Table S3.** Point Mutations between Ef.PcfG and Highly Homologous Full-Length Ll.LtrB variants in *L. lactis. (PDF 1182 kb)*
