# Supplementary material for: Recent horizontal transfer, functional adaptation and dissemination of a bacterial group II intron
Source: BMC Evol Biol. 2016 Oct 20;16:223. doi: 10.1186/s12862-016-0789-7 (PMC5072309; doi:10.1186/s12862-016-0789-7)
Supplement: Additional file 1: Table S1. — Plasmids used in this Study. Table S2. Primers used in this Study. Table S3. Point Mutations between Ef.PcfG and Highly Homologous Full-Length Ll.LtrB variants in L. lactis. (PDF 1182 kb) [file 12862_2016_789_MOESM1_ESM.pdf]

Table S1: Plasmids used in this Study

Plasmid name	Size and Antibiotic Resistance [a]	Description
pLE-Pnis-*ltrB*E1-Ll.LtrB-*ltrB*E2	13.3 kb, Cam^R	'Intron Donor'[b], with WT Ll.LtrB intron
pLE-Pnis-*pcfG*E1-Ef.PcfG-*pcfG*E2	13.1 kb, Cam^R	'Intron Donor'[b], with WT Ef.PcfG intron
pLE-Pnis-*ltrB*E1-Ef.PcfG-*ltrB*E2	13.3 kb, Cam^R	'Intron Donor'[b], with Ef.PcfG intron flanked by ltrB exons
pLE-Pnis-*pcfG*E1-Ll.LtrB-*pcfG*E2	13.1 kb, Cam^R	'Intron Donor'[b], with Ll.LtrB intron flanked by pcfG exons
pLE-Pnis-*pcfG*E1-Ef.PcfG-*pcfG*E2-Mut #1	13.1 kb, Cam^R	'Intron Donor'[b], with Ef.PcfG intron containing A→G mutation
pLE-Pnis-*pcfG*E1-Ef.PcfG-*pcfG*E2-Mut #2	13.1 kb, Cam^R	'Intron Donor'[b], with Ef.PcfG intron containing A→G mutation
pLE-Pnis-*pcfG*E1-Ef.PcfG-*pcfG*E2-Mut #3	13.1 kb, Cam^R	'Intron Donor'[b], with Ef.PcfG intron containing A→G mutation
pLE-Pnis-*pcfG*E1-Ef.PcfG-*pcfG*E2-Mut #4	13.1 kb, Cam^R	'Intron Donor'[b], with Ef.PcfG intron containing A→G mutation
pLE-Pnis-*pcfG*E1-Ef.PcfG-*pcfG*E2-Mut #5	13.1 kb, Cam^R	'Intron Donor'[b], with Ef.PcfG intron containing A→C mutation
pLE-Pnis-*pcfG*E1-Ef.PcfG-*pcfG*E2-Mut #6	13.1 kb, Cam^R	'Intron Donor'[b], with Ef.PcfG intron containing A→G mutation
pLE-Pnis-*pcfG*E1-Ef.PcfG-*pcfG*E2-Mut #7	13.1 kb, Cam^R	'Intron Donor'[b], with Ef.PcfG intron containing A→G mutation
pLE-Pnis-*pcfG*E1-Ef.PcfG-*pcfG*E2-Mut #8	13.1 kb, Cam^R	'Intron Donor'[b], with Ef.PcfG intron containing G→A mutation
pDL-*ltrB*-HS	7 kb, Spc^R	'Intron Recipient', with native Ll.LtrB Homing Site (*ltrB* gene)
pDL-*pcfG*-HS	7.3 kb, Spc^R	'Intron Recipient', with native Ef.PcfG Homing Site (*pcfG* gene)

a. Cam^R, Chloramphenicol resistance at (10µg/ml); Spc^R, Spectinomycin resistance at (300µg/ml).

b. Intron-interrupted genes (*ltrB* or *pcfG*) in 'Intron Donor' plasmids are under the control of a Nisin-inducible promoter (Pnis).

Table S2: Primers used in this Study

Primer name	Sequence (5'-3') [a]
Poisoned primer for *ltrB* exon 2	GCCAGTATAAAGATTCGTAGAAT
Poisoned primer for *pcfG* exon 2	ACCTGTTTTTAAATTGGTTGAAC
Released intron (RT)	CGATTGTCTTTAGGTAACTCAT
Released intron (PCR)	CTCTTGTTGTATGCTTTCATTG
	CTTTCCAACCGTGCTCTGTTC
Ligated exons and *pcfG*-HS fragment (RT)	AATGTCGGTTTGCTTCTCTG
Ligated exons and *pcfG*-HS fragment (PCR)	GCTTGCTCATATATTGAGATTGC
	TACGGCTTGTATTTCATGAAGCT
5' mobility junction of Ef.PcfG in *mobA* of pEF1071 (PCR)	CTGGGACAATCAAGCAACCAAA
	CTTTCCAACCGTGCTCTGTTC
3' mobility junction of Ef.PcfG in *mobA* of pEF1071 (PCR)	CTCTTGTTGTATGCTTTCATTG
	AACCACCGATAAAATTCGTCCA
Mut #1 Within Ef.PcfG (Nt 515/2492)	TTTACATGGC**A**AAGGGGTACAG
	GGGCGTTATCCTTCTCAG
Mut #2 Within Ef.PcfG (Nt 754/2492)	TACAGCGGAT**G**GCTTTAGTGAAG
	TCATCTAATATTCCTTTTGTGGAAG
Mut #3 Within Ef.PcfG (Nt 996/2492)	GCTGTCACAC**A**GCTTTGAAAAC
	TTCGTTGAGGTCTAAAACC
Mut #4 Within Ef.PcfG (Nt 1161/2492)	TTCTAAAAGC**A**GGTTATCTGGAAAAC
	ATTTATAAATCAATTGGCTCATTTTC
Mut #5 Within Ef.PcfG (Nt 1438/2492)	TAAAAGATTA**C**CCACACTCCCC
	CGTTTTTCTTGATATTCTAAAAGAAC
Mut #6 Within Ef.PcfG (Nt 1588/2492)	CTAAAAATG**G**AATTGAGTGAAGAAAAAAC
	TTGTTATGAATAAAAAGTTTTAATTGTTC
Mut #7 Within Ef.PcfG (Nt 2128/2492)	ATTTACGGAT**G**AGATAAGTCAAGC
	TGATAAGGGGATTTACATTCAC
Mut #8 Within Ef.PcfG (Nt 2195/2492)	TTAAAAGCTA**A**ATGTTGTGAATTATG
	CCTGTTTTCAAGAGTATTCC

a. Pairs of primers which were used for mutagenesis have the substituted nucleotides in bold.

Table S3: Point Mutations between Ef.PcfG and Highly Homologous Full-Length Ll.LtrB variants in *L. lactis*

Strain of Origin	Intron Location	Interrupted motif	Presence of Mut #1-#8	Additional Mutations			
L. lactis subsp. *cremoris* DPC3758 (O'Sullivan et al. 2000)	Plasmid: pAF12	Relaxase HLHN-H	Mut #2, #4, #6	Nt 1421, A→G (RT) Glu(-)→Gly(o))	Nt 1975, G→A (X) (Ala(o)→Thr(p))		
L. lactis lactis subsp. *lactis* DPC220 (Fallico et al. 2012)	Plasmid: pAH82	Relaxase HLHN-H	Mut #2, #4, #6, #8	Nt 2223, T→C (En) (Silent Mutation)			
L. lactis subsp. *lactis* 1AA59 (Ladero et al. 2015)	Chromosomal (contig)	Relaxase HLHN-H	Mut #2, #4, #5, #6, #8	Nt 59, C→G (DI)	Nt 713, A→G (RT) (Lys(+)→Arg(+))	Nt 2216, C→T (En) (Thr(p)→Ile(o))	
L. lactis subsp. *cremoris* A76 (Bolotin et al. unpublished data)	Plasmid: pQA554	Relaxase HLHN-H	Mut #2, #4, #5, #6, #8	Nt 308, ΔT (DI)	Nt 560, +G (DIVa)	Nt 1230, T→C (RT) (Silent Mutation)	Nt 2109, C→T (D) (Silent Mutation)
. *lactis* subsp. *cremoris* SK11 (Makarova et al. 2006)	Chromosomal	Complement Strand of Cell Surface Protein	Mut #2, #4, #5, #6, #8	Nt 502, C→T (DIII)	Nt 2109, C→T (D) (Silent Mutation)		
L. lactis subsp. *lactis* KLDS (Yang et al. 2013)	Chromosomal	Relaxase HLHN-H	Mut #2, #4, #5, #6, #8	N/A			
L. lactis subsp. *cremoris* TIFN6 (Erkus et al. 2013)	Chromosomal (contig)	Relaxase HLHN-H	Mut #2, #4, #5, #6, #8	N/A			
L. lactis subsp. *cremoris* TIFN5 (Erkus et al. 2013)	Chromosomal (contig)	Relaxase HLHN-H	Mut #2, #4, #5, #6, #8	N/A			
L. lactis subsp. *cremoris* HP (Lambie et al. 2014)	Chromosomal (contig)	Relaxase HLHN-H	Mut #2, #4, #5, #6, 8	N/A			
L. lactis subsp. *cremoris* FG2 (Wels et al. unpublished data)	Chromosomal (contig)	Relaxase HLHN-H	Mut #2, #4, #5, #6, 8	N/A			
L. lactis subsp. *cremoris* B40 (Wels et al. unpublished data)	Chromosomal (contig)	Relaxase HLHN-H	Mut #2, #4, #5, #6, 8	N/A			
L. lactis subsp. *cremoris* LMG6897 (Wels et al. unpublished data)	Chromosomal (contig)	Relaxase HLHN-H	Mut #2, #4, #5, #6, 8	N/A			
L. lactis subsp. *cremoris* TIFN7 (Erkus et al. 2013)	Chromosomal (contig)	Relaxase HLHN-H	Mut #2, #4, #5, #6, #8	Nt 771, ΔA (RT)	Nt 773, ΔA (RT)		
L. lactis subsp. *cremoris* TIFN1 (Erkus et al. 2013)	Chromosomal (contig)	Relaxase HLHN-H	Mut #2, #4, #5, #6, #8	Nt 174, ΔA (DI)	Nt 175, ΔT (DI)		
L. lactis subsp. *lactis* UC317 (Wels et al. unpublished data)	Chromosomal (contig)	Relaxase HLHN-H	Mut #1, #2, #4, #5, #6, #8	N/A			

L. lactis subsp. *cremoris* SK11 (Makarova et al. 2006)	Plasmid: pSK11P	Relaxase HLHN-H	Mut #1, #2, #4, #5, #6, #8	Nt 756, C→T (RT) (Silent Mutation)	Nt 1593, G→T (RT) (Leu(o)→ Phe(o))	Nt 1732, C→T (X) (Leu(o)→ Phe(o))	Nt 1958, G→A (X) (Ser(p)→ Asn(p))
L. lactis subsp. *cremoris* SK11 (Siezen et al. 2005)	Plasmid: Plasmid 3 (p3)	Relaxase HLHN-H	Mut #1, #2, #4, #5, #6, #8	Nt 756, C→T (RT) (Silent Mutation)	Nt 1593, G→T (RT) (Leu(o)→ Phe(o))	Nt 1732, C→T (X) (Leu(o)→ Phe(o))	Nt 1958, G→A (X) (Ser(p)→ Asn(p))
L. lactis subsp. *cremoris* SK110 (Wels et al. unpublished data)	Chromosomal (contig)	Relaxase HLHN-H	Mut #1, #2, #4, #5, #6, #8	Nt 756, C→T (RT) (Silent Mutation)	Nt 1593, G→T (RT) (Leu(o)→ Phe(o))	Nt 1732, C→T (X) (Leu(o)→ Phe(o))	Nt 1958, G→A (X) (Ser(p)→ Asn(p))
L. lactis subsp. *cremoris* AM2 (Wels et al. unpublished data)	Plasmid: Plasmid 3 (p3)	Relaxase HLHN-H	Mut #1, #2, #4, #5, #6, #8	Nt 756, C→T (RT) (Silent Mutation)	Nt 1593, G→T (RT) (Leu(o)→ Phe(o))	Nt 1732, C→T (X) (Leu(o)→ Phe(o))	Nt 1958, G→A (X) (Ser(p)→ Asn(p))
L. lactis subsp. *lactis* ML3 (Mills et al. 1996)	Plasmid: pRS01	Relaxase HIHN-H	Mut #1, #2, #3, #4, #5, #6, #7, #8	N/A			
L. lactis subsp. *cremoris* NZ9000 (Linares et al. 2010)	Chromosomal	Relaxase HIHN-H	Mut #1, #2, #3, #4, #5, #6, #7, #8	N/A			
L. lactis subsp. *cremoris* MG1363 (Gasson et al. 1995)	Plasmid: pFI430	Relaxase HIHN-H	Mut #1, #2, #3, #4, #5, #6, #7, #8	N/A			
L. lactis subsp. *cremoris* MG1363 (Wegmann et al. 2007)	Chromosomal	Relaxase HIHN-H	Mut #1, #2, #3, #4, #5, #6, #7, #8	N/A			
L. lactis subsp. *cremoris* NCDO763 (Wels et al. unpublished data)	Chromosomal	Relaxase HIHN-H	Mut #1, #2, #3, #4, #5, #6, #7, #8	N/A			

References:

Erkus O, de Jager VC, Spus M, van Alen-Boerrigter IJ, van Rijswijck IM, Hazelwood L, Janssen PW, van Hijum SA, Kleerebezem M, Smid EJ. 2013. Multifactorial diversity sustains microbial community stability. Isme j 7(11):2126-36.

Fallico V, Ross RP, Fitzgerald GF, McAuliffe O. 2012. Novel conjugative plasmids from the natural isolate Lactococcus lactis subspecies cremoris DPC3758: a repository of genes for the potential improvement of dairy starters. J Dairy Sci 95(7):3593-608.

Gasson M, Godon J, Pillidge C, Eaton T, Jury K, Shearman C. 1995. Characterization and exploitation of conjugation in Lactococcus lactis. International Dairy Journal 5(8):757-762.

Ladero V, Del Rio B, Linares DM, Fernandez M, Mayo B, Martin MC, Alvarez MA. 2015. Draft Genome Sequence of the Putrescine-Producing Strain Lactococcus lactis subsp. lactis 1AA59. Genome Announc 3(3).

Lambie SC, Altermann E, Leahy SC, Kelly WJ. 2014. Draft Genome Sequence of Lactococcus lactis subsp. cremoris HPT, the First Defined-Strain Dairy Starter Culture Bacterium. Genome Announc 2(2).

Linares DM, Kok J, Poolman B. 2010. Genome sequences of Lactococcus lactis MG1363 (revised) and NZ9000 and comparative physiological studies. J Bacteriol 192(21):5806-12.

Makarova K, Slesarev A, Wolf Y, Sorokin A, Mirkin B, Koonin E, Pavlov A, Pavlova N, Karamychev V, Polouchine N et al. . 2006. Comparative genomics of the lactic acid bacteria. Proc Natl Acad Sci U S A 103(42):15611-6.

Mills DA, McKay LL, Dunny GM. 1996. Splicing of a group II intron involved in the conjugative transfer of pRS01 in lactococci. J Bacteriol 178(12):3531-8.

O'Sullivan D, Twomey DP, Coffey A, Hill C, Fitzgerald GF, Ross RP. 2000. Novel type I restriction specificities through domain shuffling of HsdS subunits in Lactococcus lactis. Mol Microbiol 36(4):866-75.

Siezen RJ, Renckens B, van Swam I, Peters S, van Kranenburg R, Kleerebezem M, de Vos WM. 2005. Complete sequences of four plasmids of Lactococcus lactis subsp. cremoris SK11 reveal extensive adaptation to the dairy environment. Appl Environ Microbiol 71(12):8371-82.

Wegmann U, O'Connell-Motherway M, Zomer A, Buist G, Shearman C, Canchaya C, Ventura M, Goesmann A, Gasson MJ, Kuipers OP et al. . 2007. Complete genome sequence of the prototype lactic acid bacterium Lactococcus lactis subsp. cremoris MG1363. J Bacteriol 189(8):3256-70.

Yang X, Wang Y, Huo G. 2013. Complete Genome Sequence of Lactococcus lactis subsp. lactis KLDS4.0325. Genome Announc 1(6).
